# Antithrombotic management and outcomes of patients with atrial fibrillation treated with NOACs early at the time of market introduction: Main results from the PREFER in AF Prolongation Registry

**DOI:** 10.1007/s11739-020-02442-9

**Published:** 2020-09-21

**Authors:** Giulia Renda, Ladislav Pecen, Giuseppe Patti, Fabrizio Ricci, Dipak Kotecha, Jolanta M. Siller-Matula, Renate B. Schnabel, Rolf Wachter, Jean-Marc Sellal, Miklos Rohla, Markus Lucerna, Kurt Huber, Freek W. A. Verheugt, Jose Luis Zamorano, Bernd Brüggenjürgen, Harald Darius, Mattias Duytschaever, Jean-Yves Le Heuzey, Richard J. Schilling, Paulus Kirchhof, Raffaele De Caterina

**Affiliations:** 1 Department of Neuroscience, Imaging, and Clinical Science, G. D’Annunzio University of Chieti-Pescara, Chieti, Italy; 2 Institute of Computer Science, Academy of Sciences of the Czech Republic, Prague, Czech Republic; 3 Department of Translational Medicine, University of Eastern Piedmont and Maggiore Della Carità Hospital, Novara, Italy; 4 University of Birmingham Institute of Cardiovascular Sciences, and UHB and SWBH NHS Trusts, Birmingham, UK; 5 Department of Cardiology, Medical University of Vienna, Vienna, Austria; 6 First Department of Cardiology, Poznan University of Medical Sciences, Poznan, Poland; 7 Department for General and Interventional Cardiology, University Heart Center Hamburg, Hamburg, Germany; 8 German Center for Cardiovascular Research (DZHK) Partner Site Hamburg / Kiel/ Lübeck, Hamburg, Germany; 9 Clinic and Policlinic for Cardiology, University Hospital Leipzig, Leipzig, Germany; 10 Department of Cardiology, University Hospital of Nancy, Nancy, France; 11 Third Medical Department, Cardiology and Intensive Care Medicine, Wilhelminen Hospital, Vienna, Austria; 12 Daiichi-Sankyo Europe, Munich, Germany; 13 Third Medical Department, Cardiology and Intensive Care Medicine, Wilhelminen Hospital, and Sigmund Freud University, Medical School, Vienna, Austria; 14 Emeritus Professor of Cardiology, Amsterdam, The Netherlands; 15 Department of Cardiology, University Hospital Ramo´N Y Cajal, Madrid, Spain; 16 Institute for Health Economics, Steinbeis-University, Berlin, Germany; 17 Vivantes Hospital Neukölln, Berlin, Germany; 18 Department of Cardiology, Sint-Jan Hospital Bruges, Bruges, Belgium; 19 Georges Pompidou Hospital-René Descartes University, Paris, France; 20 London Bridge Hospital, London, UK; 21 Chair of Cardiology, University Cardiology Division, University of Pisa, Pisa University Hospital, Pisa, and Fondazione VillaSerena Per La Ricerca, Città Sant’Angelo, Pescara, Italy; 22 DZHK (German Centre for Cardiovascular Research), Partner site, Göttingen, Germany

**Keywords:** Atrial fibrillation, Anticoagulants, NOAC, Registry, Major cardiac or cerebrovascular events, Bleeding

## Abstract

The management of patients with atrial fibrillation (AF) has rapidly changed with increasing use of non-vitamin K antagonist oral anticoagulants (NOACs) and changes in the use of rhythm control therapy. The prevention of thromboembolic events European Registry in Atrial Fibrillation Prolongation Registry (PREFER Prolongation) enrolled consecutive patients with AF on NOACs between 2014 and 2016 in a multicentre, prospective, observational study with one-year follow-up, focusing on the time of introduction of NOACs. Overall, 3783 patients were enrolled, with follow-up information available in 3223 (85%). Mean age was 72.2 ± 9.4 years, 40% were women, mean CHA_2_DS_2_VASc score was 3.4 ± 1.6, and 2587 (88.6%) had a CHA_2_DS_2_VASc score ≥ 2. Rivaroxaban was used in half of patients, and dabigatran and apixaban were used in about a quarter of patients each; edoxaban was not available for use in Europe at the time. Major cardiovascular event rate was low: serious events occurred in 74 patients (84 events, 2%), including 24 strokes (1%), 62 major bleeds (2%), of which 30 were life-threatening (1%) and 3 intracranial (0.1%), and 28 acute coronary syndromes (1%). Mortality was 2%. Antiarrhythmic drugs were used in about 50% of patients, catheter ablation in 5%. Adverse events were low in this contemporary European cohort of unselected AF patients treated with NOACs already at the time of their first introduction, despite high thromboembolic risk.

**Electronic supplementary material:**

The online version of this article (10.1007/s11739-020-02442-9) contains supplementary material, which is available to authorized users.

## Introduction

The non-vitamin K antagonist oral anticoagulants (NOACs) are increasingly used as an alternative to vitamin K antagonists (VKAs) to prevent strokes in patients with atrial fibrillation (AF) [1–4]. They include the direct thrombin inhibitor dabigatran, and the direct factor Xa inhibitors rivaroxaban, apixaban and edoxaban. When compared with warfarin in phase III randomized clinical trials [5–8], NOACs showed a consistently favourable benefit-risk profile across a wide range of patients, with lower mortality and a lower rate of intracranial haemorrhage than patients randomized to VKA [9, 10].

The aim of the PREFER in AF Prolongation study was to collect information on unselected patients with AF treated with NOACs at the beginning of their widespread introduction in Europe [11].

## Methods

PREFER in AF Prolongation was a multinational, multicentre, prospective, observational study with a baseline visit at the time of patient enrolment and a 1-year follow-up visit, with comparable data sets and methods to the prior PREFER in AF [12]. It was conducted in the same seven European countries as PREFER in AF registry, Austria, France, Germany, Italy, Spain, Switzerland, United Kingdom (UK), and in two additional countries, Belgium and the Netherlands. The baseline enrolment occurred between June 2014 and May 2015 and patients underwent follow-up visit after 1 year (12 ± 1 months, namely 364 ± 33 days, after baseline visit); therefore, follow-up was concluded on June 2016, just before the publication of the current ESC guidelines on the management of AF [13].

Patients were included if they were at least 18 years of age, provided written informed consent for participation in the Registry, had a documented diagnosis of AF by ECG within the prior 12 months, and were treated with an oral anticoagulant. 10% of patients continued from PREFER in AF, of these around half were treated with VKAs and the remaining treated with NOACs. All newly enrolled patients reported in this analysis were treated with a NOAC, except edoxaban, that was not available for use in Europe at the time of PREFER in AF Prolongation registry. No explicit exclusion criteria were defined to encourage consecutive enrolment apart from patients with mechanical valve replacements or at least moderate mitral stenosis which were not eligible. Follow-up was performed one year after enrolment. Following types of clinical events were recorded during 1-year follow-up: ischemic stroke, transient ischemic attack (TIA), acute coronary syndrome, ST-elevation myocardial infarction (STEMI), non-ST-elevation myocardial infarction (NSTEMI), unstable angina pectoris, stent insertion, coronary bypass surgery, chronic heart insufficiency, reduced left ventricular ejection fraction, arterial embolism, gastrointestinal bleeding, intracerebral bleeding, other life-threatening or major bleeding, venous thromboembolism event, and pulmonary thromboembolic event. Between these, major adverse cardiac and cerebrovascular events (MACCE) were evaluated. Data were captured through an electronic case report form (eCRF) including plausibility checks for the entered variables. The study management was overseen by a scientific steering committee, executed by a contract research organization (SSS International Clinical Research GmbH, Munich, Germany). The sponsor of the study was Daiichi-Sankyo Europe GmbH, Munich.

### Statistical analysis

All variables collected in the eCRF at baseline and follow-up, and all derived parameters were used in the statistical analysis. Statistical analysis was performed only on patients with complete case data from baseline and follow-up visits, including those who died during the 1-year follow-up (for these patients we know the reason of death and information about drugs prescribed before death).

Binary, categorical, and ordinal parameters were summarized by means of absolute and percentage numbers within the various categories. Percentages were calculated from available results (there were few missing values, due to not entirely completed electronic case report forms). Numerical data were summarized by means of standard statistics (i.e., number of available data, number of missing data, mean, standard deviation, minimum, median, maximum, and lower and upper quartiles). Differences between follow-up and baseline data were evaluated by the Wilcoxon test for continuous variables and by the Chi-squared test for discrete variables. Mantel–Haenszel Chi-squared test was also used to assess the trend for categorical versus ordinal variables. Statistical significance was evaluated by a univariate logistic regression model. A two-tailed *p*-value of < 0.05 was considered statistically significant.

The statistical analysis was performed using SAS v. 9.4 (SAS Institute Inc., Cary, USA).

## Results

### Patient characteristics and clinical presentation of atrial fibrillation

**Fig. 1:**
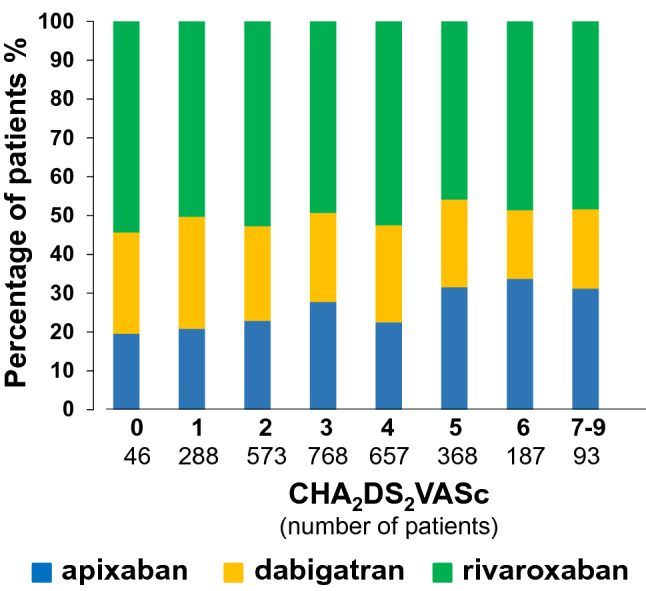
Frequency of NOAC prescription according to thromboembolic risk. Percentage of patients treated with apixaban (blue), dabigatran (yellow), and rivaroxaban (green) according to the CHA_2_DS_2_VASc score. Patient number for each category is reported in brackets

Overall, 3783 patients were enrolled into the PREFER in AF Prolongation registry. One-year follow-up was available in 3223 patients (85.2%). At enrolment, the mean age was 72.2 ± 9.4 years, and 59.9% were male. Clinical characteristics of the population, including risk factors and comorbidities, are reported in Table 1. Compared with baseline, at follow-up, there were a higher number of patients with permanent AF (40.8% vs 31.4% at baseline, *P* < 0.0001) and lower number with persistent AF (12.3% vs 23.6% at baseline, *P* < 0.0001) (OS Fig. 1). At baseline, 2707 patients (84.1%) had symptomatic AF (defined by European Heart Rhythm Association, EHRA, score ≥ II), and 2329 patients (74.1%) were symptomatic at follow-up (*P* < 0.0001). Fatigue and dyspnoea were the most frequent symptoms; less frequent were palpitations, dizziness, chest pain, and anxiety. Severe symptoms, reflected by EHRA scores of III or IV, were less common at follow-up compared with baseline (EHRA IV = 10.1% vs 15.0%; III = 21.2% vs 28.0%; *P* < 0.0001), while more patients had occasional symptoms or were asymptomatic at follow-up compared with baseline (EHRA II = 42.9% vs 41.2%; I = 25.9% vs 15.9%; *P* < 0.0001). The mean EHRA score decreased from 2.42 ± 0.93 at baseline to 2.16 ± 0.92 at 1-year follow-up visit *P* < 0.0001).

**Table 1:** Main clinical characteristics of the population (*N* = 3223)

Age years, mean ± SD	72.2 ± 9.4
Female *n* (%)	1292 (40.1)
Arterial hypertension *n* (%)	2466 (76.7)
Diabetes mellitus (DM) *n* (%)	745 (23.1)
Insulin therapy *n* (%)	178 (24.0% of DM, 5.5% of pts)
Obesity (BMI > 30) *n* (%)	933 (29.2)
Chronic obstructive pulmonary disease *n* (%)	278 (8.6)
Current smoking *n* (%)	221 (7.0)
Dyslipidaemia *n* (%)	1310 (41.4)
Hyperthyroidism *n* (%)	128 (4.0)
Chronic renal insufficiency *n* (%)	630 (19.7)
Chronic hepatic disease *n* (%)	31 (1.0)
Major previous GI/ cerebrovascular/other bleeding events *n* (%)	121 (3.8)
Heart valve dysfunction *n* (%)	1193 (37.3)
Heart valve replacement *n* (%)	51 (4.3% of HVD, 1.6% of pts)
Type: biological valve *n* (%)	49 (96.1% of HVR)
Coronary heart disease (CHD) *n* (%)	648 (20.3)
Stent insertion *n* (%)	345 (54.2% of CHD, 10.8% of pts)
Type: Bare-metal stent *n* (%)	174 (52.7% of CHD)
Type: Drug-eluting stent *n* (%)	156 (47.3% of CHD)
Previous MI *n* (%)	279 (8.7)
Previous ischemic stroke *n* (%)	383 (11.9)
Previous TIA *n* (%)	326 (10.2)
Previous other ischemic-thromboembolic event *n* (%)	196 (6.1)
Previous ischemic stroke/TIA/other ischemic-thromboembolic event *n* (%)	530 (16.5)
Thromboembolic events *n* (%)	87 (2.7)
Chronic heart insufficiency *n* (%)	664 (20.7)
Reduced left ventricular ejection fraction *n* (%)	432 (13.7)
Chronic heart insufficiency/reduced left ventricular ejection fraction *n* (%)	799 (24.8)
Peripheral Arterial Disease (PAD) *n* (%)	105 (3.3)
Prevalent cardiovascular disease (CHD, PAD, MI) *n* (%)	696 (21.9)
HAS-BLED < 3 *n* (%)	1942 (73.6)
Age > 65 *n* (%)	2534 (78.6)
Age ≥ 75 *n* (%)	1394 (43.3)
Uncontrolled hypertension (sBP > 160 mmHg) *n* (%)	1251 (47.4)
Concomitant use of drugs (such as antiplatelet agents, NSAIDs) *n* (%)	268 (8.4)
Excess alcohol use *n* (%)	118 (3.7)

Percentages were calculated from the available results. *BMI *body mass index, *GI *gastrointestinal, *MI *myocardial infarction, *NSAIDs *nonsteroidal anti-inflammatory drugs, *TIA *transient ischemic attack

### Thromboembolic and bleeding risk

Thromboembolic risk, evaluated by CHA_2_DS_2_VASc score, was high at both baseline and follow-up (mean CHA_2_DS_2_VASc: 3.36 ± 1.57 and 3.40 ± 1.56, respectively). The majority of patients had a CHA_2_DS_2_VASc score ≥ 2 (88.6% at baseline and 89.5% at follow-up). Modifiable and non-modifiable risk factors for bleeding (including concomitant use of drugs such as antiplatelet agents and nonsteroidal anti-inflammatory drugs, uncontrolled hypertension, excess alcohol use, chronic renal insufficiency, chronic hepatic disease, age > 65 and ≥ 75, history of previous stroke, history of major bleeding) are reported in Table 1. Particularly, antiplatelet drugs (mainly aspirin and/or clopidogrel) were used in 392 patients, (12%) in addition to NOAC at baseline [338 patient (10.5%) were also treated with a single antiplatelet agent and 54 patients (1.7%) with dual antiplatelet agents]. Combination therapy was, however, less frequent at follow-up [180 patients, 5.7%, *P* < 0.0001; 155 patients (4.9%) were treated with one and 25 patients (0.79%) were treated with two antiplatelet agents] (see Table 2).

**Table 2:** Distribution of NOACs, alone or in combination with antiplatelets, at baseline and follow-up

Drug	Baseline *n* (%)	Follow-up* n (%)	Follow-up vs baseline *P*
NOACs	3223 (100.0)	3149 (100.0)	NA
NOACs monotherapy	2831 (87.84)	2969 (94.28)	< 0.0001
NOACs + antiplatelets	392 (12.16)	180 (5.72)	< 0.0001
*Type of NOAC*			
Dabigatran	797 (24.73)	738 (23.44)	0.2277
Rivaroxaban	1667 (51.72)	1613 (51.22)	0.6901
Apixaban	842 (26.12)	879 (27.91)	0.1078
*Type of antiplatelet*			
Aspirin	327 (10.15)	142 (4.51)	< 0.0001
Clopidogrel	104 (3.23)	58 (1.84)	0.0004
Prasugrel	4 (0.12)	1 (0.03)	0.1881
Ticagrelor	2 (0.06)	3 (0.10)	0.6359
Single antiplatelet therapy	338 (10.5)	155 (4.9)	< 0.0001
Double antiplatelet therapy	54 (1.67)	25 (0.79)	0.0015

^*^Patients who died and were withdrawn from NOACs during 1-year FU were excluded from the FU column. Statistically significant *P* values are in bold

### Stroke prevention therapy

Rivaroxaban was used by 1667 patients (52%) treated with NOACs, compared with dabigatran and apixaban, which were used by 797 (25%) and 842 (26%) patients, respectively, with minimal variations between baseline and follow-up visit (Table 2); in 83 patients (3%), two types of NOAC were reported at baseline (usually intake of one of them was stopped shortly after baseline visit).

**Fig. 2:**
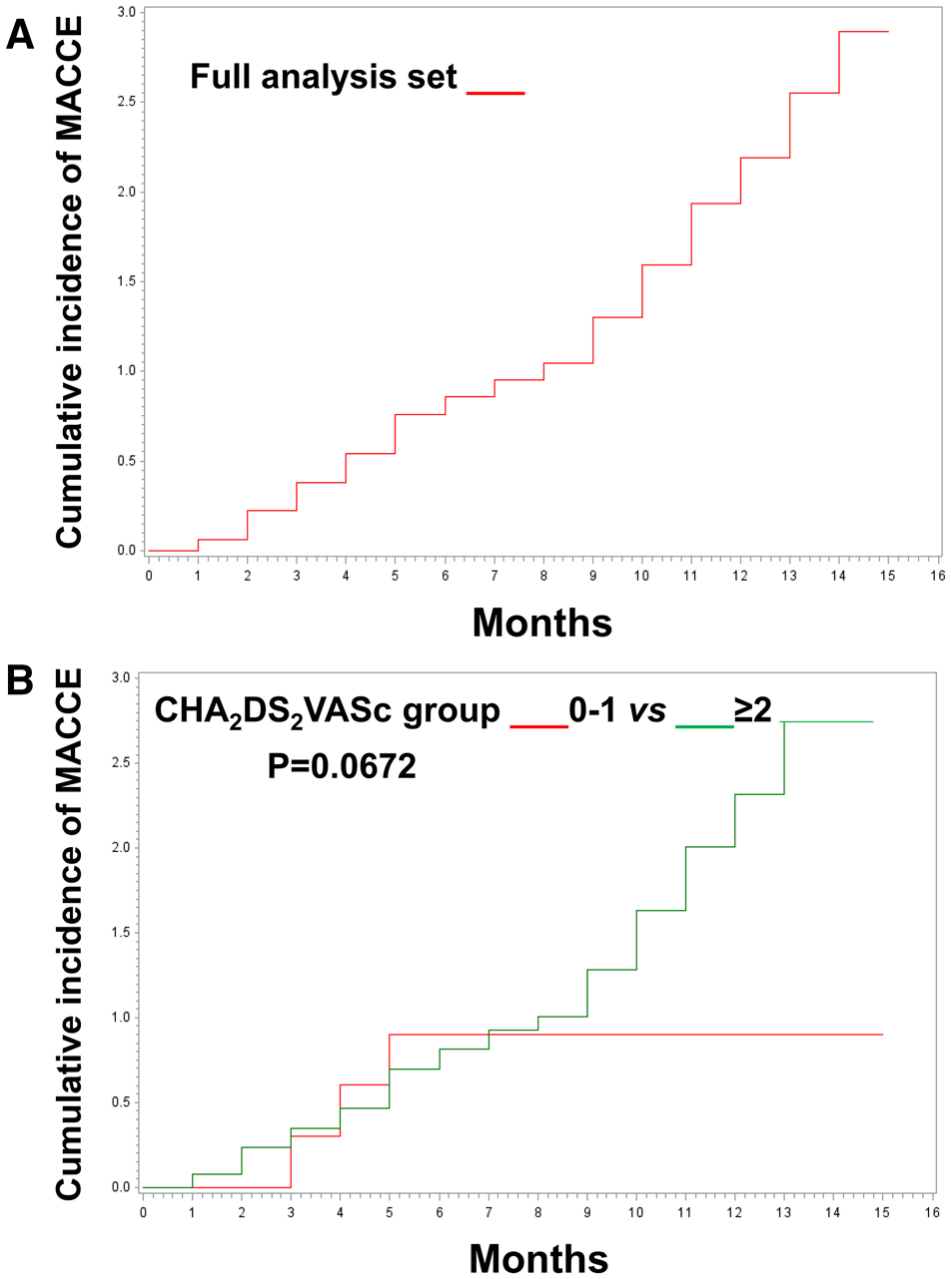
Cumulative proportion of MACCE during the observation period. Panel A shows full analysis set. Panel B shows the incidence of MACCE according to the CHA_2_DS_2_VASc score ≥ 2 (green line) vs 0 or 1 (red line)

The different distribution of NOAC use in the nine European countries was reported in OS Table 1. Out of 3223 patients, 5 (0.16%) discontinued a NOAC; 117 patients (3.6%) switched from one NOAC to another during the 1-year follow-up (OS Fig. 2); 29 patients (25% of switchers) switched to rivaroxaban (mainly from dabigatran); 68 patients (58%) switched to apixaban (mainly from rivaroxaban); and 20 patients (17%) switched to dabigatran (mainly from rivaroxaban); 48 patients (6.0%) treated with dabigatran, 55 (3.3%) treated with rivaroxaban and 14 (1.7%) treated with apixaban switched to another NOAC (*p* = 0.0617; OS Fig. 2). The reasons given for switching in these 117 patients were adverse drug reaction (26 patients, 22.2%), inconvenience/non-compliance (20 patients, 17.1%), lack of efficacy (mainly related to the occurrence of cardiovascular events in 13 patients, 11.1%), drug-drug interaction (6 patients, 5.1%), minor surgery (5 patients, 4.3%) and high variability of response (2 patients, 1.7%). Drug changes occurred without a documented reason in 76 patients (65%), and 31 patients (1%) had more than one reason.

Apixaban was used slightly more often with increasing CHA_2_DS_2_VASc score *P* = 0.0004); while dabigatran was prescribed nominally less often with increasing CHA_2_DS_2_VASc score (*P* = 0.0389) (Fig. 1). There was no detectable correlation between CHA_2_DS_2_VASc score and rivaroxaban prescription (*P* = 0.2165) (Fig. 1). Similar associations were observed with increasing HAS-BLED scores (data not shown).

### Clinical events

A total of 468 clinical events occurred in 348 patients (10.8%) between baseline and follow-up: of these, 24 were strokes (0.8%), 62 (2.0%) major bleeding of which three were intracerebral bleeding (0.1%) and 37 other life-threatening bleeding events (1.2%), 28 were acute coronary syndromes (0.9%) and 2 (0.1%) were arterial embolisms (OS Table 2 shows the main clinical events that occurred between baseline and follow-up). No significant difference (*P* = 0.1922) was found in major bleeding between patients not treated with antiplatelet drugs (1.8%) and patients treated with one (3.04%; HR = 1.71, 95% CI = 0.86–3.40) or two antiplatelet agents (3.85%; HR = 2.18, 95% CI = 0.52–9.20).

Mortality was 2.1% (69 deaths were reported out of 3223 patients); cardiovascular (CV)-related death comprised 40 (58.0%) of all deaths (1.2% of all patients). The most common fatal event was unspecified cardiovascular death (20 deaths), followed by death due to heart failure (10 deaths) and cancer (8 deaths) (OS Table 3). A total of 84 major adverse cardiac and cerebrovascular events (MACCE) occurred in 74 patients (2.3%; Fig. 2a): in addition to the strokes and life-threatening bleeding, they included 28 acute coronary syndromes (0.9%).

**Fig. 3:**
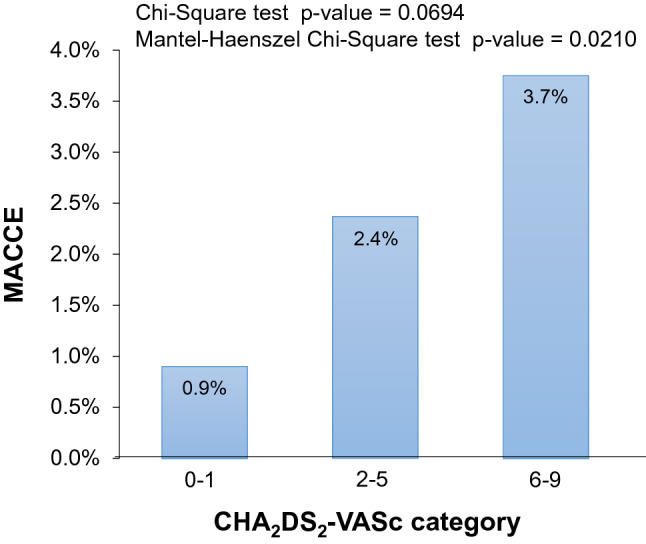
CHA_2_DS_2_VASc score in patients presenting MACCE compared with those not presenting. Error bars show standard deviation

Overall, the incidence of MACCE appeared higher in patients with a CHA_2_DS_2_VASc score ≥ 2 (2.5%) compared with patients with a score of 0 or 1 (0.9%; *P* = 0.0672), although there was no difference in MACCE incidence between CHA_2_DS_2_VASc 0–1 vs ≥ 2 during first 6 months (Fig. 2b). The highest incidence of MACCE is for a CHA_2_DS_2_VASc score = 7, followed by a CHA_2_DS_2_VASc score = 6. There are a very low number of patients with CHA_2_DS_2_VASc 8–9, and no MACCE events (OS Table 4). Figure 3 shows the incidence of MACCE according to CHA_2_DS_2_VASc category. CHA_2_DS_2_VASc score was different between patients with and without events (3.74 ± 1.51 with MACCE vs. 3.33 ± 1.56 without MACCE, *p* = 0.0361).

### Rate and rhythm control therapy

Control of heart rate, as assessed by the resting ECG, was generally good, both for asymptomatic and symptomatic patients; more patients had adequate rate control (heart rate 60–100/min) at follow-up compared with baseline (81.8% versus 78.4%; *P* = 0.0009). Rhythm control procedures, i.e. cardioversion, pharmacological or electrical cardioversion or AF ablation, were performed in 356 patients (11.3%) during follow-up, more often used in the year before enrolment than in the year after enrolment (OS Table 5). No relationship was observed between the type of NOAC and cardioversion or ablation (data not shown). Antiarrhythmic drugs at baseline were used in 1597 (49.6%). The most commonly used antiarrhythmic drug was amiodarone (16.3% of patients), followed by flecainide 9.7%, sotalol 5.0%, propafenone 1.4%, dronedarone 1.2% and quinidine 0.6%; 15.4% of patients were treated with other antiarrhythmic, not specified, drugs. At the follow-up visit, antiarrhythmic drugs were used in 43.1% of patients (1391 patients; *P* < 0.0001 vs baseline); again, amiodarone was the most used drug (13.4% of patients), followed by flecainide (9.2%), sotalol (5.1%), dronedarone (1.0%), propafenone (0.9%) and quinidine (0.4%); 13.1% of patients were treated with other antiarrhythmic, not specified, drugs.

## Discussion

The PREFER in AF Prolongation study provides valuable information on the characteristics and outcomes of patients treated with NOACs in Europe in 2014 and 2015, coinciding with the time of introduction of these at that time novel drugs in the therapeutic armamentarium. Patient characteristics (mean age 72.5 years, 60% male) were comparable with those of PREFER in AF [12] and other registries [14–16]; therefore, this cohort can be considered representative for the management of AF at that time. Thromboembolic risk was high, as expected in a cohort of anticoagulated patients with AF, and comparable to the phase III trials [5–8] and to other AF registries [12, 14–16]. This is notable as initial reports of the use of NOACs found that they were often in “low-risk” patients. Bleeding risk as estimated by HAS-BLED score was relatively low, and lower at 1-year follow-up, reflecting less unnecessary antiplatelet treatment [17] and potentially reduction of other modifiable bleeding risk factors. Importantly, the use of combination therapy with NOACs plus antiplatelets decreased by half from baseline to follow-up, this is probably explained by the interruption of a dual antiplatelet therapy started prior to enrolment and completed after prescribed period, or to a higher attention to guideline indications, particularly in combination therapy, sometimes inappropriately used in stable coronary artery disease ^17^. Rivaroxaban was prescribed in half of the patients, while dabigatran and apixaban were used in about a quarter of patients, respectively. Since edoxaban was not available for use in Europe at the time of enrolment in the PREFER in AF Prolongation registry, we have no data about this drug in this registry. The incidence of switching from one NOAC to another was low (3.6%) during the 1-year follow-up, without a clear pattern between the different agents. Voluntary switch was the main reason for changing.

Stroke rate (24 events, 0.8%) and major cardiovascular event rate (2.3%, 84 events in 74 patients) were low and seemed related to thromboembolic risk factors. MACCE rate was nominally lower than that observed in PREFER in AF, enrolled in a similar registry only 2 years earlier (237 events in 217 patients, 3.4%) [12]. This may reflect a slightly lower risk of the population enrolled in the PREFER in AF Prolongation compared with that enrolled in PREFER in AF. Most likely, the lower rate of events was due to a different anticoagulant treatment: all patients newly enrolled in the PREFER in AF Prolongation were treated with a NOAC, while only 6% of patients enrolled in PREFER in AF were treated with a NOAC, 77% were treated with a VKA and the others with antiplatelet agents or no antithrombotic drug.

Accordingly, mortality was low (2.1%), and CV-related mortality was 57.5% of the total mortality. Although the overall incidence of MACCE appeared higher in patients with a CHA_2_DS_2_VASc score ≥ 2 compared with patients with a score of 0 or 1 at 1-year follow-up, this difference became more evident after 6 months, probably due to the absence of events in patients with CHA_2_DS_2_VASc 0–1 in this period. Overall, the registry demonstrates, in a real-life setting, the low rates of thromboembolic and bleeding events occurring in well-treated patients with AF. This is in agreement with data from other real-life studies, overall reporting a lower incidence of adverse events compared with randomized clinical trial. The rate of events may be different between registries due to different inclusion criteria, study design, period of data collection, duration of follow-up, regional contribution and participating physicians.

The proportion of patients with permanent AF increased during follow-up, reflecting the progressive nature of AF. The use of rhythm control therapy, mainly antiarrhythmic drugs, but also AF ablation, was lower compared to other reports [18], and decreased from baseline to follow-up. At the same time, patients were less symptomatic at follow-up than at baseline, illustrating the changing nature of symptoms in patients with atrial fibrillation, probably reflecting the higher number of patients with permanent AF.

## Study limitations

This analysis from the PREFER in AF Prolongation registry provides a snapshot of AF patients treated with NOACs in nine European countries, and the follow-up data shows the one-year evolution compared with baseline. However, about 15% of patients were lost to follow-up, and information about events that occurred the year before baseline and during follow-up was not always complete. Therefore, statistical analysis was performed only on patients with complete case data from baseline and follow-up visits, including dead patients which data were completely recorded.

Inherent to other similar registries, selection bias cannot be ruled out at the centre or patient level. Moreover, here we considered only patients treated with NOACs, but we could not obtain either information about previous anticoagulant treatment (time of NOAC initiation or time of switching from VKAs) or about the dosages of each drug. Finally, due to the observational nature of the study, the effects of different NOACs could not be compared.

## Conclusions

This registry of European patients at the beginning of the NOAC era found low event rates in AF patients treated with NOACs, despite high thromboembolic risk. This provides reassurance regarding the use of these agents in “real-life” conditions already at the beginning of the NOAC era. Treatment patterns had already changed compared to earlier reports, including a lower use of combination therapy with antiplatelet agents, and the improved management of modifiable bleeding risk factors over time, reflecting the rapidly shifting reality of AF management with the introduction of the NOACs.

## Electronic supplementary material

Below is the link to the electronic supplementary material.

Supplementary file1 (DOCX 168 kb)
