# Supplementary material for: Antithrombotic management and outcomes of patients with atrial fibrillation treated with NOACs early at the time of market introduction: Main results from the PREFER in AF Prolongation Registry
Source: Intern Emerg Med. 2020 Sep 21;16(3):591–9. doi: 10.1007/s11739-020-02442-9 (PMC8049932; doi:10.1007/s11739-020-02442-9)
Supplement: Supplementary file 1 — Supplementary file1 (DOCX 168 kb) [file 11739_2020_2442_MOESM1_ESM.docx]

**Antithrombotic management and outcomes of patients with atrial fibrillation treated with NOACs early at the time of market introduction:**

**Main results from the PREFER in AF Prolongation Registry**

**ONLINE SUPPLEMENTAL MATERIAL**

**OS TABLES**

| **OS Table 1.** Distribution of NOACs in the 9 European Countries | | | | |
| --- | --- | --- | --- | --- |
| **COUNTRY** | **Total** | **Apixaban** | **Dabigatran** | **Rivaroxaban** |
|  | **n** | **%** | **%** | **%** |
| **AUSTRIA** | 4 | 0.0 | 25.0 | 75.0 |
| **BELGIUM** | 95 | 39.0 | 15.8 | 45.2 |
| **SWITZERLAND** | 9 | 0.0 | 0.0 | 100.0 |
| **GERMANY** | 1014 | 27.1 | 14.0 | 58.9 |
| **SPAIN** | 290 | 37.9 | 25.9 | 36.2 |
| **FRANCE** | 732 | 16.4 | 21.0 | 62.6 |
| **UK** | 306 | 32.7 | 24.2 | 43.1 |
| **ITALY** | 536 | 24.1 | 41.6 | 34.3 |
| **NETHERLANDS** | 227 | 29.5 | 31.7 | 38.8 |
| **TOTAL** | 3213* | 26.0 | 24.0 | 50.0 |
| * for 10 patients type of NOAC was not given | | | | |

| **OS Table 2.** Main clinical events in 12 months between baseline and follow-up | |
| --- | --- |
|  |  |
| **EVENTS** | **Patients* N (%)** |
| Stroke | 24 (0.8) |
| Acute coronary syndrome | 28 (0.9) |
| Arterial embolism | 2 (0.1) |
| TIA | 27 (0.9) |
| Any major bleeding | 62 (2.0) |
| Intracranial hemorrhage | 3 (0.1) |
| Bleeding in critical organ other than brain | 24 (0.8) |
| Any other Bleedings | 37 (1.2) |
| Hospitalization due to Clinical Events | 224 (7.0) |
| Hospitalization due to Bleeding Events | 80 (2.5) |
| Hospitalization due to Clinical or Bleeding Events | 271 (8.4) |
|  |  |
| *patients with at least 1 event |  |

| **OS Table 3.** Causes of death in 12 months between baseline and follow-up | |
| --- | --- |
|  |  |
| **EVENTS** | **n (%)** |
| Total | 69 (2.1) |
| Cardiovascular | 40 (1.2) |
| Cardiavascular unspecified | 20 (0.6) |
| Heart failure | 10 (0.3) |
| Reduced left ventricular ejection fraction | 3 (0.09) |
| Myocardial infarction | 2 (0.06) |
| Stroke | 0 (0.0) |
| Arterial embolism | 1 (0.03) |
| Pulmonar & Venous embolism | 1 (0.03) |
| Sudden death | 3 (0.09) |
| Non cardiovascular | 29 (0.9) |
| Cancer | 8 (0.2) |
| Infection | 6 (0.2) |
| Major bleeding | 2 (0.06) |
| Neurological | 2 (0.06) |
| Injury/accident | 2 (0.06) |
| Other/unspecified non cardiovascular | 9 (0.3) |

| **OS Table 4. Incidence of MACCE by CHA_2_DS_2_-VASc score** | | | | | | | | | | | | |
| --- | --- | --- | --- | --- | --- | --- | --- | --- | --- | --- | --- | --- |
| **CHA_2_DS_2_-VASc** | | | | | | | | | | | | |
| **MACCE** | | **0** | **1** | **2** | **3** | **4** | **5** | **6** | **7** | **8** | **9** | **Total** |
| No | Number | 46 | 283 | 556 | 740 | 619 | 349 | 174 | 63 | 17 | 3 | 2850 |
|  | % | 100 | 98.95 | 97.72 | 98.01 | 97.17 | 97.49 | 96.13 | 95.45 | 100 | 100 |  |
| Yes | Number | 0 | 3 | 13 | 15 | 18 | 9 | 7 | 3 | 0 | 0 | 68 |
|  | % | 0 | 1.05 | 2.28 | 1.99 | 2.83 | 2.51 | 3.87 | 4.55 | 0 | 0 |  |
| Total | Number | 46 | 286 | 569 | 755 | 637 | 358 | 181 | 66 | 17 | 3 | 2918 |
| Frequency Missing = 305 | | | | | | | | | | | | |

| **OS** **Table 5**. Procedures of cardioversion and ablation | | | |
| --- | --- | --- | --- |
|  |  |  |  |
| **PROCEDURES** | **Baseline (n=3223)** | **Follow-up (n=3149*)** | **Follow-up vs baseline** |
|  | **n (%)** | **n (%)** | **P** |
| **Pharmacological Cardioversion** | 372 (11.54%) | 90 (2.85%) | **<0.0001** |
| **Electrical Cardioversion** | 575 (17.84%) | 204 (6.47%) | **<0.0001** |
| **Ablation** | 169 (5.24%) | 119 (3.78%) | **0.0049** |
|  |  |  |  |
| *for 69 patients which died and for other 5 this info in missing at follow-up | | | |
| Statistically significant P values are in bold | |  |  |

**OS FIGURES**

**
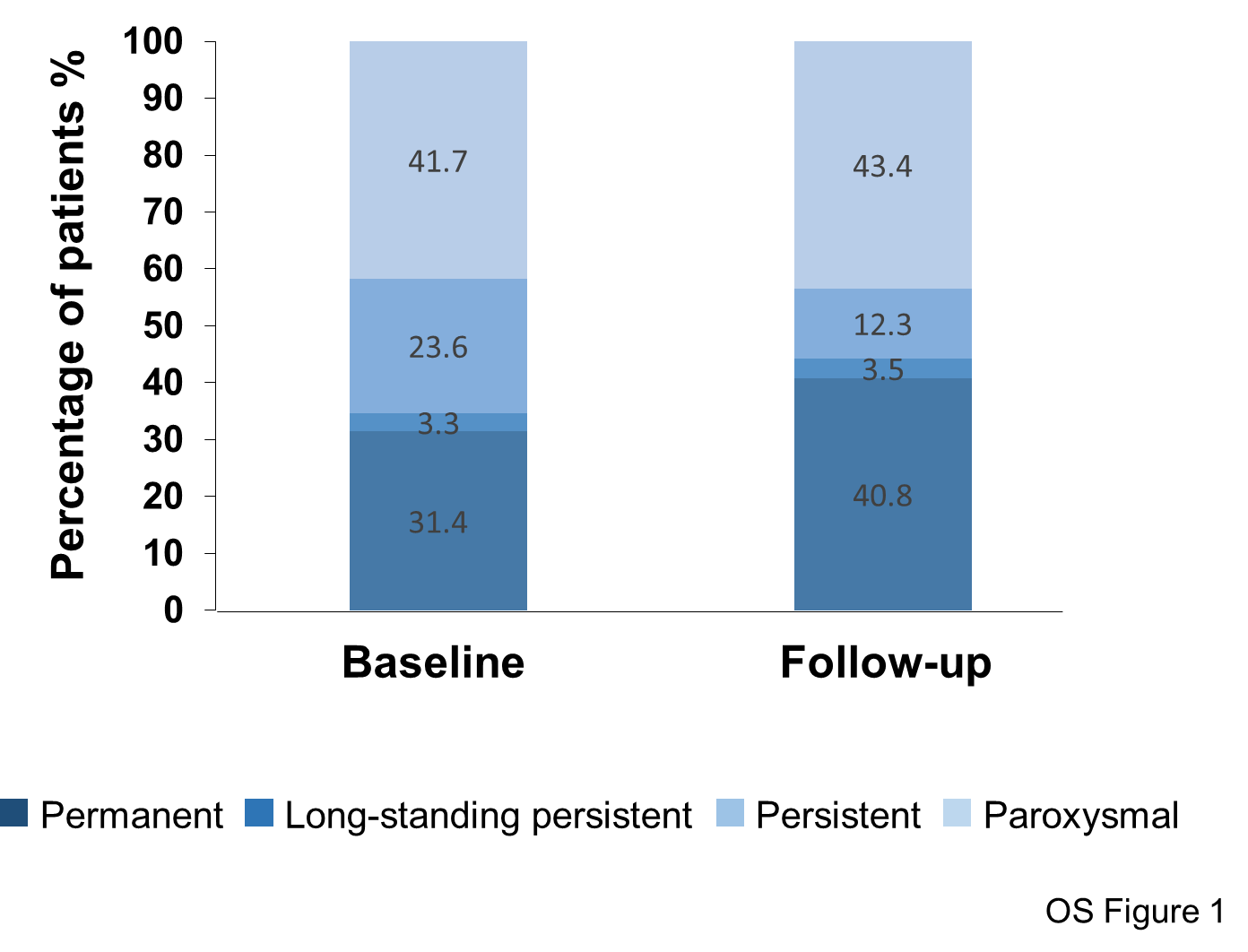
**

**
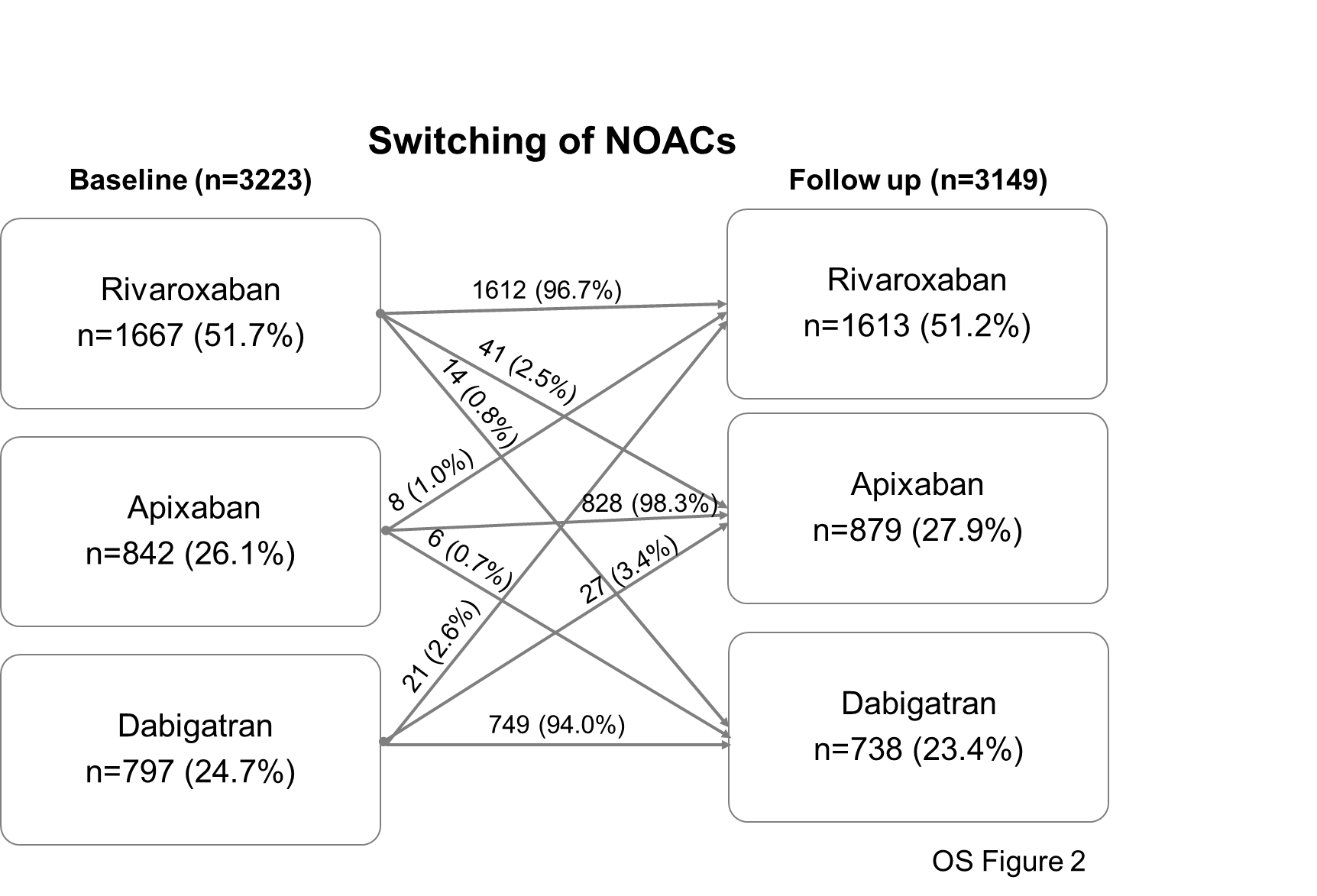
**

**LEGENDS TO OS FIGURES**

**OS Figure 1.** Clinical presentation of AF at baseline and follow-up. The darker blue represents permanent AF, the lighter paroxysmal AF.

**OS Figure 2.** Switching of NOACs from baseline to follow up. Percentages of patients switching from a NOAC to another were reported above the arrows. 5 patients who were withdrawn from NOACs during 1 year follow up were not presented in this figure.
